# Influence of Selective Laser Melting Additive Manufacturing Parameters in Inconel 718 Superalloy

**DOI:** 10.3390/ma15041362

**Published:** 2022-02-12

**Authors:** Nikolaos Kladovasilakis, Paschalis Charalampous, Konstantinos Tsongas, Ioannis Kostavelis, Dimitrios Tzovaras, Dimitrios Tzetzis

**Affiliations:** 1 Centre for Research and Technology Hellas, Information Technologies Institute (CERTH/ITI), 57001 Thessaloniki, Greece; n.kladovasilakis@ihu.edu.gr (N.K.); pcharalampous@iti.gr (P.C.); gkostave@iti.gr (I.K.); Dimitrios.Tzovaras@iti.gr (D.T.); 2 Digital Manufacturing and Materials Characterization Laboratory, School of Science and Technology, International Hellenic University, 57001 Thessaloniki, Greece; k.tsongas@ihu.edu.gr

**Keywords:** selective laser melting (SLM), Inconel 718, roughness, mechanical behavior, tensile testing, regression models

## Abstract

Selective laser melting (SLM) is one of the most reliable and efficient procedures for Metal Additive Manufacturing (AM) due to the capability to produce components with high standards in terms of dimensional accuracy, surface finish, and mechanical behavior. In the past years, the SLM process has been utilized for direct manufacturing of fully functional mechanical parts in various industries, such as aeronautics and automotive. Hence, it is essential to investigate the SLM procedure for the most commonly used metals and alloys. The current paper focuses on the impact of crucial process-related parameters on the final quality of parts constructed with the Inconel 718 superalloy. Utilizing the SLM process and the Inconel 718 powder, several samples were fabricated using various values on critical AM parameters, and their mechanical behavior as well as their surface finish were examined. The investigated parameters were the laser power, the scan speed, the spot size, and their output Volumetric Energy Density (VED), which were applied on each specimen. The feedstock material was inspected using Scanning Electron Microscopy (SEM), Energy-dispersive X-ray spectroscopy (EDX) analysis, and Particle-size distribution (PSD) measurements in order to classify the quality of the raw material. The surface roughness of each specimen was evaluated via multi-focus imaging, and the mechanical performance was quantified utilizing quasi-static uniaxial tensile and nanoindentation experiments. Finally, regression-based models were developed in order to interpret the behavior of the AM part’s quality depending on the process-related parameters.

## 1. Introduction

Over the last decade, Additive Manufacturing (AM) has been developed into one of the most rapidly evolved and studied manufacturing process [1]. Aside from rapid prototyping, AM technologies are capable of producing complex structures without geometric constraints, enabling the utilization of advanced design methods such as topology optimization and generative design [2,3]. Thus, researchers are focused on the optimization of AM procedures in order to fabricate fully functional parts with high quality. Nowadays, there are eight distinct AM categories according to the international standards [4,5]. The selective laser melting (SLM) additive manufacturing technique belongs to Powder Bed Fusion (PBF) AM classification and is one of the most reliable methods for producing metal components. A plethora of industries have investigated the possibility of employing the SLM technique to fabricate mechanical parts with advanced design features [6,7]. SLM is an AM technique that utilizes a laser beam to fully melt the feedstock material in the form of a metal powder to manufacture a product. The powder spreading mechanism along the building platform is achieved via a coater and the laser beam melts the powder particles according to the component’s cross-section, creating in that way a thin layer of the overall structure. Subsequently, the building platform descends for a layer height and the procedure is repeated until the entire part is built [8]. This technique is employed for a wide range of metal and alloy materials such as stainless steels, titanium alloys, superalloys, etc. [9]. Due to the fact that this procedure melts the feedstock material to form the desired structure, there is an optimal manufacturing strategy for every material, which results in optimal part’s quality. However, each quality factor, such as surface roughness and mechanical performance, exhibits different behavior depending on the applied process-related parameters. Thus, it is essential to establish a process map that demonstrates the performance of the quality factors depending on the applied parameters in order to facilitate the trade-offs that have to be employed to manufacture a high-quality metal component.

The quality of a metal 3D-printed product could be evaluated by measuring a series of factors, such as the dimensional accuracy, the quality of the part’s surface, and its mechanical response. Currently, several studies have investigated the quality of parts manufactured with the SLM technique using as the construction material stainless steel, aluminum alloy, titanium alloys, etc. [10,11,12]. According to existing studies [13,14], process-related parameters possess a major impact on the dimensional accuracy and the surface finish of the manufactured object. More specifically, Calignano et.al. [13] utilized the Taguchi method as well as fractional factorial experiments (FFE) in order to highlight the effect of the laser power and the scan speed on the surface roughness of aluminum parts. Fotovatti et.al. [14] concluded that the lower layer thickness and hatching distance are, the higher surface quality would be achieved. In addition, several studies have shown that process-related parameters, such as the laser power, scan speed, and spot size affect the melting procedure with the regulation of the molten pool size, which has a direct impact on the surface quality [15,16,17]. Furthermore, the influence of process-related conditions on the mechanical behavior of a metal AM product depends on the extracted relative density of the part and the existence of discontinuities inside the part’s volume. These malfunctions and defects occurred due to two different phenomena that could be developed during the melting process: the lack of fusion and the keyhole effect [18]. The lack of fusion occurs in situations where the applied volumetric energy density (VED) is not sufficient enough to fully melt the feedstock material and the already built material near to the surface, disabling the formation of a uniform molten pool. On the other hand, the keyholing effect occurs in situations where there is a surplus of applied VED on the metal powder, leading to the formation of regions with entrapped gases or vapors in the internal structure of the manufactured part. According to existing literature [18,19], metal vaporization could occur from constituents with a low melting point inside the alloy. These two mechanisms are strongly dependent on the applied thermal energy that is measured via the volumetric energy density [19,20]:

(1) $$\mathrm{VED}=\frac{P}{V\cdot h\cdot \delta }$$

where P is the laser power, V is the scan speed, h is the hatching distance, and δ is the layer height. Moreover, it is worth mentioning that existing papers concluded that materials that are resistant to high thermal strains such as SS316 and Inconel 625 are the most susceptible to present lack of fusion defects [21,22].

**Figure 1:**
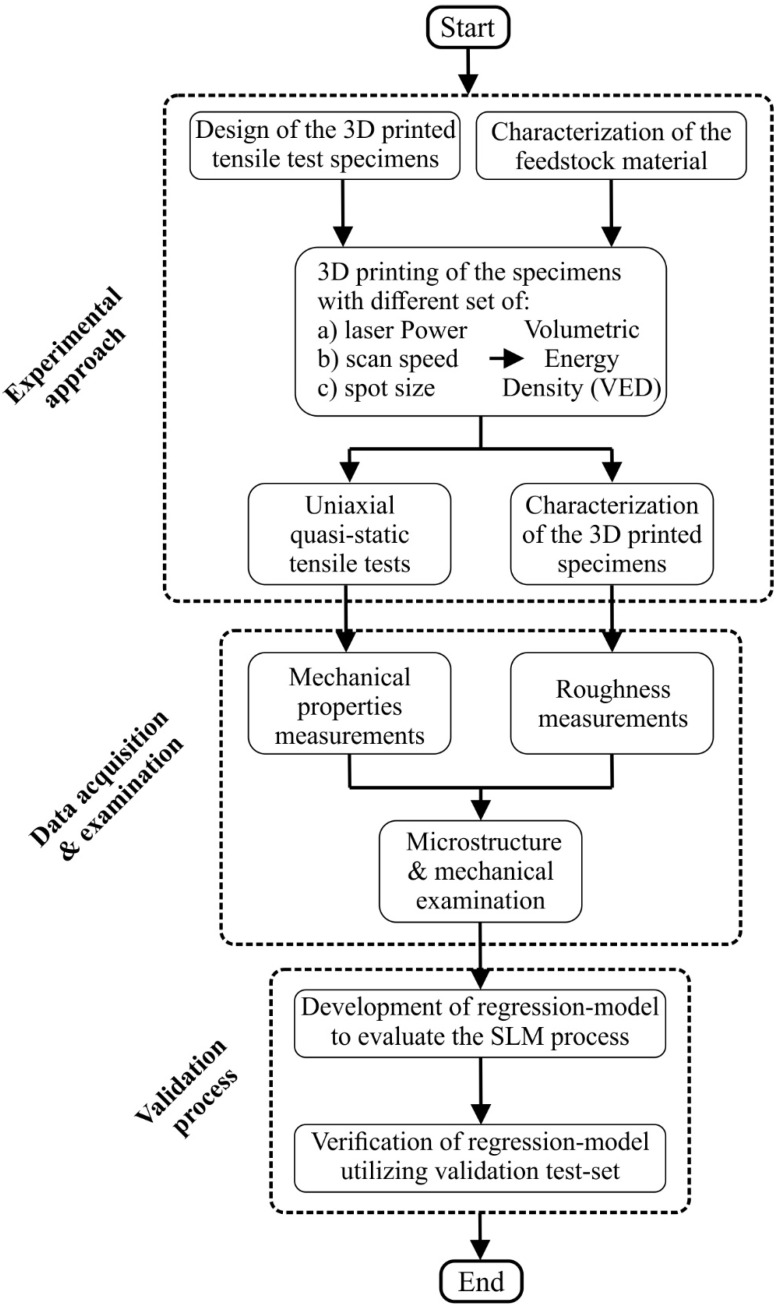
Flowchart of the current research.

A plethora of metal materials has been investigated in the existing literature using different process-related parameters by measuring the quality of the produced parts in terms of dimensional accuracy, surface quality, and mechanical performance. It must be noted that for distinct feedstock materials, different values on the manufacturing parameters are applied; yet the main defect mechanisms remain the same. Furthermore, numerous studies have been performed for additive manufacturing with Inconel 718 as a construction material [23,24,25,26,27]. These studies have investigated the influence of process-related parameters on microstructures, texture, anisotropy, and mechanical properties of Inconel 718 AM parts. However, the majority of the studies examined a limited set of parameters without extracting a continuous behavior about the material regarding the applied process parameters. Therefore, the current study aims to correlate various critical printing parameters to the quality of SLM manufactured Inconel 718 products using regression-based models as statistical tools. In the present work, a systematic investigation of the surface characteristics of the produced samples was studied as well as their mechanical behavior. More particular, various printing parameters sets were applied in order to point out their effect on the quality of the Inconel 718 additively manufactured components. The results of the experimental procedures were inspected using SEM as well as EDX analysis, and the mechanical performance was examined via nanoindentation and tensile experiments. In addition, four polynomial regression models were developed, performing an analytical investigation of the relationship between the dependent (manufacturing conditions) and independent variables (surface roughness, elastic modulus, yield stress, and ultimate tensile stress). Figure 1 portrays the applied methodology of the present study.

## 2. Materials and Methods

### 2.1. Material & Surface Characterization

In the current study, the examined material was the superalloy Nickel alloy 718, also known as Inconel 718, in the form of powder (OC Oerlikon, Freienbach, Switzerland). The characterization of the feedstock powder is an essential task for metal AM procedures in order to achieve the optimal quality on the 3D printed product [8,28]. Hereupon, a Scanning Electron Microscope (SEM), was employed for the material characterization process. More specifically, the Phenom ProX Desktop SEM (ThermoFisher Scientific, Massachusetts, MA, USA) was used to examine the powder’s morphological characteristics. Furthermore, utilizing the SEM’s specialized modules, a Particle Size Distribution (PSD) analysis, as well as an Energy-Dispersive X-ray Spectroscopy (EDX), were performed on the feedstock powder, extracting that way the size of the powder particles and the exact chemical composition of the superalloy respectively. Moreover, SEM was also applied in order to examine the microstructure of the 3D-printed specimens and the fracture surface that emerged after the tensile experiments. In addition, the stereoscope Leica DMS 1000 (Leica Microsystems GmbH, Wetzlar, Germany) with a plan-apochromatic objective and magnification up to 300x coupled with the MountainsLab^®^ (Digital Surf, Besançon, France) software were employed to acquire high-resolution multi-focus images and to measure the surface roughness for each manufactured specimen with an accuracy of ±1 μm according to the ISO 4287 [29].

### 2.2. Modeling and AM Process

**Figure 2:**
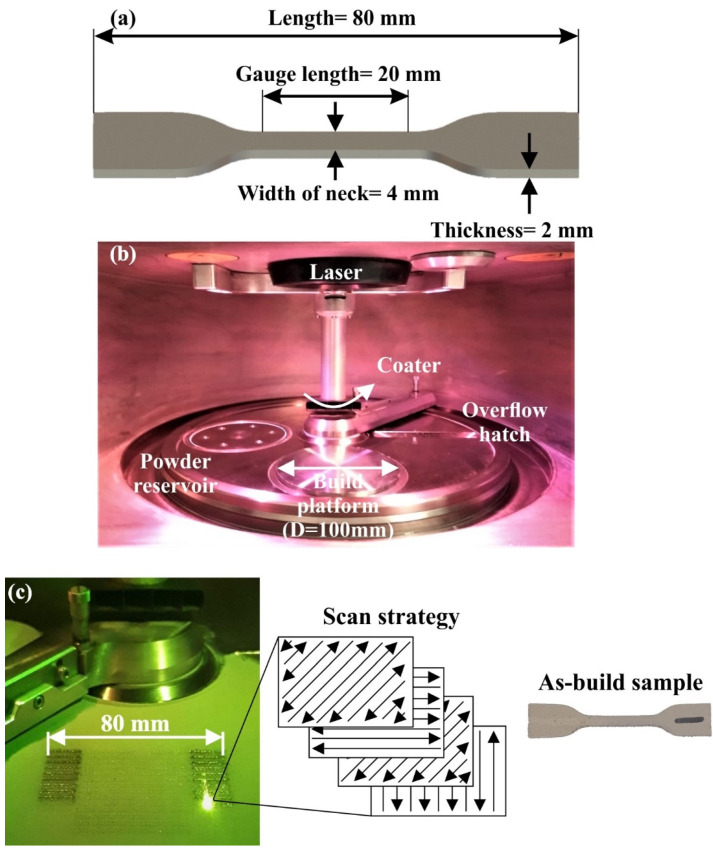
(**a**) Basic dimensions of the tensile specimens; (**b**) building chamber of the SLM 3D printer; and (**c**) the SLM 3D printing process coupled with scan strategy with a front view of as-build sample.

In order to investigate the influence of process-related parameters on the surface quality and the mechanical performance of a metal 3D-printed part, tensile test specimens were designed according to ISO 527 [30]. More specifically, the 5A type for tensile specimens was designed in SolidWorks™ (Dassault Systèmes SE, Vélizy-Villacoublay, France) software, and it was selected due to its relatively small length (≥75 mm), facilitating the manufacturing process. Figure 2a depicts the basic dimensions of the designed tensile specimens. The selective laser melting (SLM) technique was employed utilizing the ORLAS CREATOR (Coherent Inc., Santa Clara, CA, USA) metal 3D printer. ORLAS CREATOR utilizes a continuous Yb-fiber laser beam with 250 W maximum power coupled with a wavelength of 1067 nm to successfully melt the metal powder. Furthermore, the maximum printing accuracy of the applied metal 3D printer reaches up 25 μm at the vertical direction (layer height), and the minimum hatching distance was applied with value of 40 μm. It is necessary to note that the process was performed without applying any additional heat. Figure 2b,c exhibit the building chamber in idle mode and printing mode respectively. Moreover, as scan strategy, the 45° rotation of the scan vector was utilized, as is illustrated in Figure 2c, due to the fact that it minimizes the anisotropy [31]. It is worth mentioning that the XZY orientation was chosen according to the ASTM standardization [32] and as a result of the trade-off between the optimal mechanical response and minimum internal stresses [33]. Figure 2c also presents the front view of the as-build sample in order to clarify the build orientation and the laser paths. In the context of this research, three distinct process-related parameters were examined, namely the laser power, scan speed, and spot size (laser beam diameter).

The laser power varied from 120 to 160 W, the scan speed ranged between 900–1100 mm/s, and the spot size between 45–75 μm. To construct a valid and reliable data set, several experiments were conducted, altering the examined printing conditions. In total, 37 distinct SLM printing experiments were conducted, with repeatability of three samples, adjusting in each one a different printing parameter. More particular, the 27 different combinations (with ID: P1-P27) were fabricated and used as a training data set, as is exhibited in the upper section of Table 1. Furthermore, in the bottom part of Table 1, the experimental procedures with ID: P28-P37 are documented corresponding to the validation set in order to examine the performance of the developed regression models.

### 2.3. Mechanical Testing

Prior to the tensile experiments, the nanoindentation process was conducted on each 3D-printed Inconel 718 sample, extracting its microhardness and the elastic modulus depending on the applied printing conditions. The Dynamic Ultra Micro Hardness Tester DUH-211S (Shimadzu Copr., Tokyo, Japan) was employed for the nanoindentation process equipped with a Berkovich diamond indenter using a 100 nm tip radius and a resolution of 0.196 μN. The indenter penetrated the test surface with a specified load of 200 mΝ. The Oliver–Pharr formula was utilized in order to compute the elastic modulus and the microhardness [34]. It is worth noting that multiple (at least 10 measurements) nanoindentation tests were performed per sample scattered along their polished surfaces. In addition, the 3D-printed test specimens were examined under quasi-static uniaxial tensile loading at room temperature utilizing a universal testing machine (M500-50AT Testometric Company, Rochdale, UK) equipped with a 50 kN load cell. At least three tensile coupons were used. All the specimens were tested according to the international standards for tensile testing, and the strain rate was selected as 5 mm/min [35].

### 2.4. Regression-Based Model

In general, regression models are employed in order to identify the relationship between a dependent output and one or more independent inputs. It consists of one of the most widespread statistical tools that is applied in several scientific fields. In the present study, the polynomial regression model was utilized to estimate the dependence of several variables such as the arithmetic mean roughness, Young’s modulus, ultimate tensile strength, and yield strength with the printing parameters of the SLM AM process. In the first step, the regression coefficients of the developed models were computed using the least square method. After the fitting of the data, the models were evaluated employing some common indicators as well as with a validation set. These data were not considered during the development of the regression models; hence the validation set behaves like ‘unseen data’. It must be noted that the Scikit-learn library [36] was used to construct the appropriate regression models, predicting the mechanical behavior as well as the surface finish of the manufactured metal components using the SLM technology.

In particular, second-order polynomial regression models were developed to evaluate the relationship of the input data (laser power, scanning speed, and spot size) with each of the output parameter (roughness, Young’s modulus, ultimate tensile strength, and yield strength). In general, a regression model describes the relationship between n independent input variables x_1_, x_2_,…, x_n_ and one single dependent output value y as follows [37]:

(2) $$y=b_{0}+\overset{n}{\underset{i=1}{\sum }}b_{i}x_{i}+\overset{\;}{\underset{i<m}{\sum }}b_{\mathrm{im}}x_{i}x_{m}+\overset{n\;}{\underset{i=1}{\sum }}b_{\mathrm{ii}}x_{i}^{2}+e\;$$

where b_0_ is the constant term, bi are the coefficients of the first-order terms, b_im_ are the interaction coefficients of the model, and b_ii_ are the quadratic coefficients of the regression model. Finally, the parameter e is the error term of the prediction model that incorporates all the possible deviations from the real measured value. It should be noted that the estimation of the regression coefficients is achieved via the least square method. The goal of the above-mentioned method is the calculation of the model’s coefficients (b_0_, b_i_, b_im_, and b_ii_), minimizing the sum of the squared errors. A more comprehensive description of the polynomial regression method is available on the following references [36,37].

## 3. Results and Discussion

### 3.1. Characterization of Inconel 718 Powder

All figures and tables should be cited in the main text as Figure 1, Table 1, etc. In general, the manufactured parts using the SLM AM technology are highly influenced by the quality of the feedstock material. According to the comprehensive review of Vock et.al [28], particle size distribution affects both the surface roughness and the mechanical properties due to the changes in the flowability of the powder and the existence of air inside the powder’s mixture. Furthermore, the chemical composition of the material demonstrates an essential role in final quality, as a compromised chemical composition of the material could lead to several defects on the 3D-printed components. Thus, an extensive characterization of the powder was performed employing three non-destructive quality control methods, namely the EDX analysis, PSD analysis, and SEM imaging. In Table 2, the chemical composition of the powder is listed according to the manufacturer (OC Oerlikon AG, Freienbach, Switzerland).

**Figure 3:**
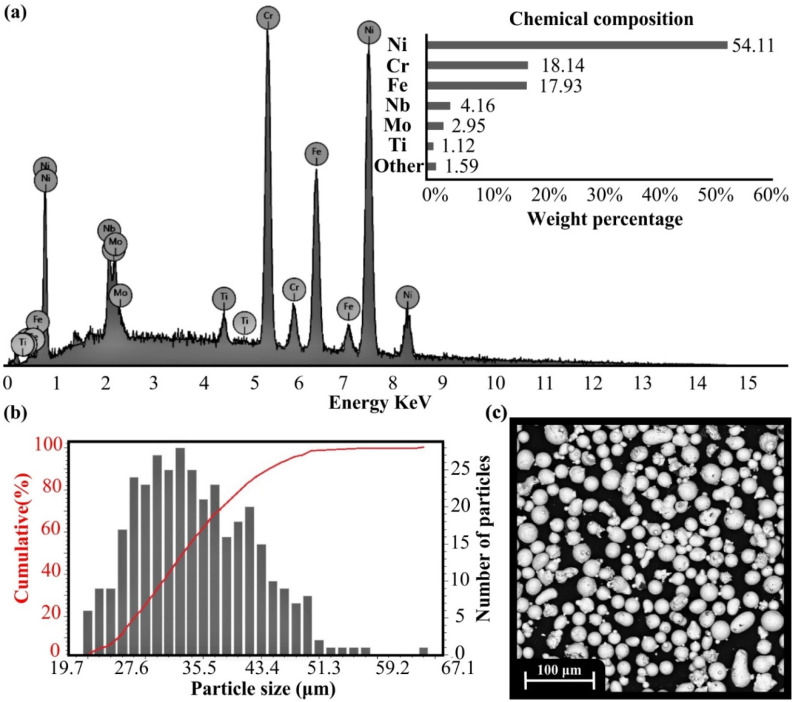
(**a**) EDX analysis and chemical composition; (**b**) PSD analysis; (**c**) SEM image for Inconel 718 powder.

EDX analysis extracted the chemical composition of the Inconel 718 powder, as shown in Figure 3a. The two chemical compositions are almost identical without any significant differences. It is worth mentioning that multiple measurements were observed and evaluated. Figure 3b presents the PSD analysis for the powder’s samples, where the PSD demonstrates a typical positive skew distribution (shifted to the left), indicating a fine particle distribution with a percent of 10% to be smaller than 24.44 μm (D10). In addition, the mean particle size was at 32.34 μm (D50) and the 90% of the particles possessed a size lower than 46.16 μm (D90). These results are derived from the red curve in Figure 3b, which represents the cumulative percentage of the powder’s particles depending on their size. The abovementioned results are slightly increased compared to the manufacturer values, as it is indicated in the bottom part of Table 2. Nevertheless, the PSD values remain very fine and suitable for fabricating high-quality AM metal parts. The microstructure and morphology of Inconel’s 718 metal powder are depicted in Figure 3c. According to the SEM analysis, the majority of the particles possess a uniform and circular shape. However, the experiments revealed that there exist particles with irregular shapes and slightly increased sizes, which may affect the quality of AM parts.

### 3.2. Surface Characterization

**Figure 4:**
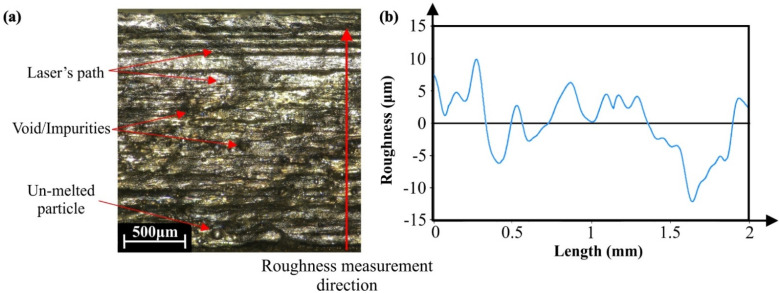
Indicatives: (**a**) Multi-focus image and (**b**) roughness profile of the P8 sample.

The characterization of the surface finish was performed utilizing the Leica DMS 1000 stereoscope and the MountainsLab^®^ software. During the surface characterization process, multi-focus images were acquired and analyzed in order to measure the arithmetic mean roughness (Ra) for each manufactured specimen. Figure 4a depicts an indicative multi-focus image from a metal 3D-printed specimen using the SLM technology. In this image, the paths of the laser beam are visible, as well as structural defects such as un-melted particles, voids, and impurities. Figure 4b presents an indicative profile of the attained surface for a metal 3D-printed component. As is shown in the figure, the direction of measurement was perpendicular to the laser path due to the higher roughness. The roughness profile possesses peaks and dales that range between −15 μm and 15 μm. It is worth mentioning that in order to obtain accurate and reliable results, five measurements per specimen were conducted. However, higher peaks were formed at the edge of the samples that exceed the 30 μm, due to the intense shrinkage effect that occurred during the AM procedure. The roughness profile resembles a characteristic roughness profile of sand-cast metal components [38], which is an expected behavior due to the melting process that is applied in the SLM AM technique. Furthermore, depending on the examined specimen, the roughness profile could fluctuate more due to the developed defects generated from the metal 3D-printed process.

**Figure 5:**
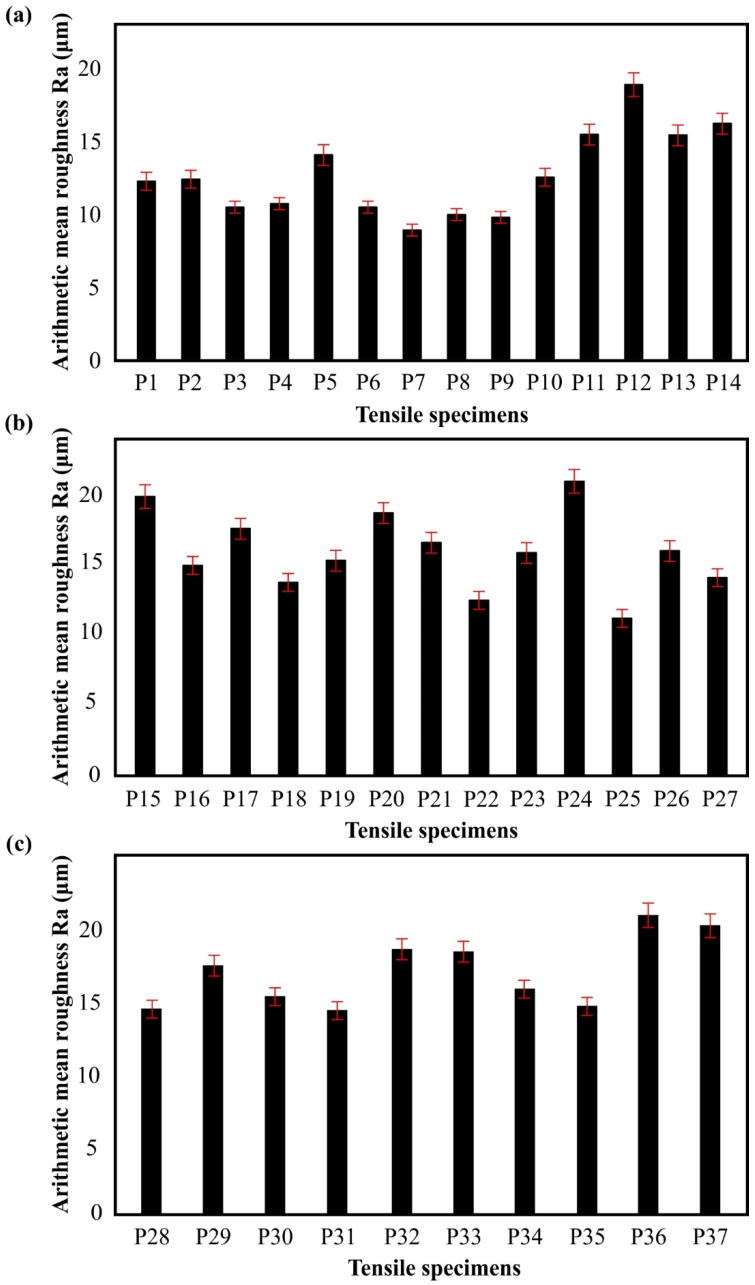
Experimental results for the arithmetic mean roughness Ra of the samples’s top surface measured at y-axis: (**a**) P1-P14, (**b**) P15-P27, and (**c**) P28-P37.

Figure 5 contains data concerning the arithmetic mean roughness Ra for all the examined specimens, where its value ranges between 9.10 μm to 21.12 μm for test specimens of P7 and P36, respectively. In general, low values of Ra (around 10 μm) were observed for the parts that were manufactured with sufficient energy density coupled with low scan speed, like the P7 and P8 (Figure 5a), due to the formation of uniform melting pools of the feedstock material [13]. Furthermore, in situations where the energy density was higher or lower than the average value (141 J/mm^3^), the developed moderate surface tension of the molten pools resulted in an increase of the surface roughness values ranging between 12 μm and 17 μm, such as at specimens P18 and P22 (Figure 5b), which are acceptable values for the SLM process. However, for extreme values of the applied energy density and scan speed, the Ra parameter was measured above 18 μm, like in the case of specimen P24, where that behavior was a result of the intense surface tension and the high cooling rate of the molten pool. In addition, it is worth mentioning that the spot size possesses a crucial role in the final surface quality, as high laser beam diameters could uniformly diffuse the thermal energy of the laser beam, achieving uniform molten pools with mild surface tension [8]. A characteristic example of the abovementioned trend is noticeable in experiment P9, where high energy density and high spot size diffused the energy surplus and thus formed an external surface with an exceptional Ra of 10 μm. To sum up, there is a strong dependency between the surface quality and the examined process parameters that are analytically evaluated in Section 3.4.

### 3.3. Microstructure Characterization and Mechanical Testing

**Figure 6:**
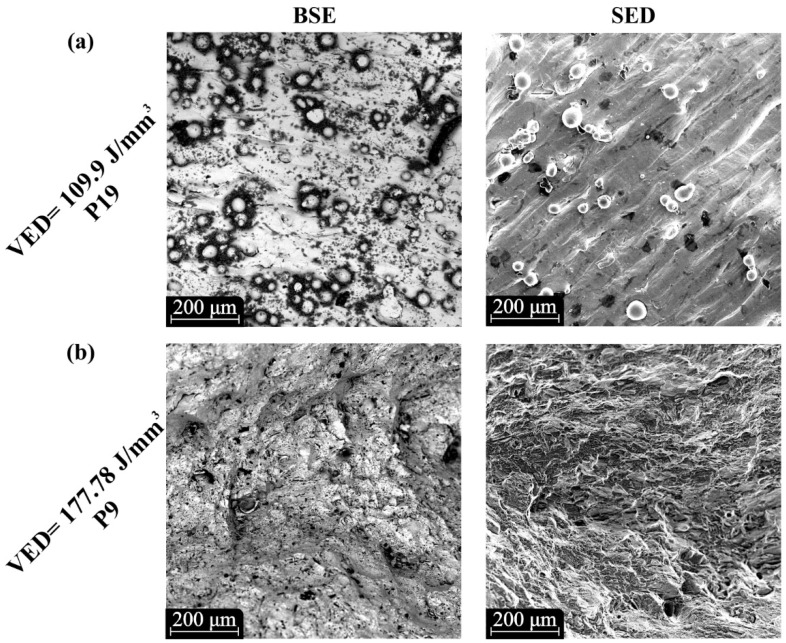
SEM images for the front surface of the specimens: (**a**) 3D printing strategy with low energy density (<110 J/mm^3^); (**b**) 3D printing strategy with high energy density (>160 J/mm^3^).

The examination of metal 3D-printed parts’ microstructure was performed with SEM analysis and imaging that were acquired utilizing both Backscattered Electrons (BSE) detector and Secondary Electron Detector (SED) in order to enhance the accuracy of the analysis. According to Figure 6a, the specimens that were built with low energy density had extensive regions of un-melted powder particles, which had been sintered with the rest of the structure. This behavior resulted in irregularities at the external surfaces and discontinuities in specimens’ microstructure, compromising the mechanical performance of the manufactured products. More specifically, in Figure 6a, SEM images of specimens manufactured with energy density below 110 J/mm^3^ are portrayed with visible un-melted particles. On the other hand, as the energy density was increased, this phenomenon deteriorated and in situations where the energy density surpassed the 160 J/mm^3^, the un-melted particles disappeared, as is depicted in Figure 6b. The absence of un-melted regions led to smoother surfaces and improvement of the mechanical properties. However, increased energy densities close to 180 J/mm^3^ provoked keyhole effects, deteriorating the mechanical behavior of the printed parts.

**Figure 7:**
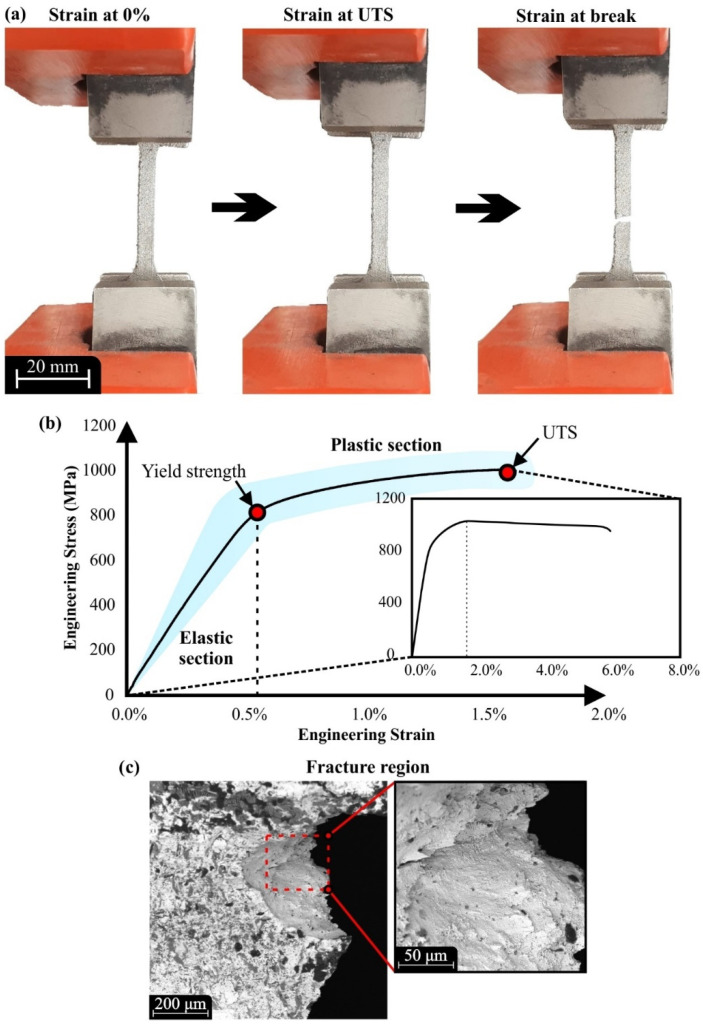
(**a**) Tensile loading at strain 0%, at peak stress, and at break; (**b**) stress–stain diagram for tensile loading for all test specimens (the shaded regions represent all the experimental data); (**c**) indicative SEM image from a fracture region.

The mechanical testing of the specimens was performed in two stages using a nanoindentation technique and a quasi-static uniaxial tensile loading. Nanoindentation experiments were employed to measure the microhardness and the elastic modulus on the polished surfaces of the specimens and report the bulk properties of the material. In addition, the tensile testing was conducted to verify the elastic modulus measurements, as well as to acquire the yield strength and the ultimate tensile strength (UTS). Moreover, the stress–strain diagrams were extracted and the mechanical response of the specimens depending on the investigated 3D printing parameters was evaluated. Figure 7a portrays characteristic steps of the conducted tensile experiments in crucial stages of the testing process. In detail, the left image shows the specimen on the testing machine without applying any load. The middle part illustrates the specimen at the maximum strain receiving the maximum tensile load (UTS), and the right part of the figure depicts the specimen right after the fracture, which occurred without necking effect. It is worth mentioning that the wrought and cast Inconel 718 tensile specimens revealed limited necking effect, according to the existing literature [38]. Furthermore, Figure 7b presents the limits of the extracted stress–strain curves that were derived from all the experimental data. Moreover, a mean curve of all the examined specimens is illustrated coupled with the confidence intervals that were extracted from the experimental data for each stage of the tensile testing. It must be noted that the percentage difference between the highest to the lowest values of mechanical properties, namely the elastic modulus, yield stress, and UTS, did not exceed 25%. It is worth noting that the elongation at UTS for all specimens ranged between 1.5% and 1.7% of strain, which indicates high stiffness of the produced metal 3D-printed components. According to the literature [24], the fracture of specimens is between 7−25% of strain.” Figure 7c illustrates a fracture region with a superimposed image of the specimen after the uniaxial tensile testing. More particularly, this state reveals the rough microstructure of the fracture surface that is alike with a dimpled structure. This rough morphology potentially occurred due to intergranular failure of the test specimen [39,40], and similar tensile fracture surfaces were observed in the existing literature [41].

In Table 3, the values of the examined mechanical properties, as well as the energy density and the properties of wrought Inconel 718 samples, which have the highest impact on the resulted mechanical properties, are reported for each fabricated specimen. It is worth mentioning that the standard deviation for all examined mechanical properties reached between 2–3%. The indentation hardness and elastic modulus of the SLM printed parts were determined based on the calculation method of Oliver and Pharr [34,42]. The hardness (H) can be calculated as a function of the maximum penetration depth of the indentation:

(3) $$H=\frac{P_{\max}}{A}$$

where P_max_ is the maximum applied load measured at the maximum depth of penetration (h_max_), and A is the projected contact area between the indenter and the film. For a perfect Berkovich indenter, A can be expressed as a function of the contact indentation depth hf as:

(4) $$A=3\sqrt{3}h_{f}^{2}\tan^{2}65=23.96h_{f\;}^{2}$$

The contact indentation, h_f_, can be determined from the following expression:

(5) $$h_{f}=h_{\max}-\varepsilon \frac{P_{\max}}{S}$$

where ε is a geometric constant ε = 0.75 for a pyramidal indenter, and S is the contact stiffness that can be determined as the slope of the unloading curve at the maximum loading point, i.e.,

(6) $$S={\left(\frac{\mathrm{dP}}{\mathrm{dh}}\right)}_{h=h_{\max}}$$

The reduced elastic modulus E_r_ is given by:

(7) $$E_{r}=\frac{S}{2\beta }\sqrt{\frac{\pi }{A}}$$

where β is a constant that depends on the geometry of the indenter. For the applied Berkovich indenter, the parameter β is equal to 1.034. The specimen elastic modulus (E_s_) can then be calculated as:

(8) $$\frac{1}{{Ε}_{r}}=\frac{1-\upsilon_{s}^{2}}{E_{s}}+\frac{1-\upsilon_{i}^{2}}{E_{i}}$$

where Ε_i,s_ and ν_i,s_ are the elastic modulus and the Poisson’s ratio for the indenter and the specimen, respectively. Moreover, for a diamond indenter, E_i_ is 1140 GPa and ν_i_ is 0.07. The specimen’s hardness H and elastic modulus E_s_ were computed from the set of equations documented above.

The specimens P18, P15, and P24 demonstrated the highest hardness values that could be attributed to the higher applied laser power, which enabled the efficient melt and fusion of the Inconel powder particles. In general, it was observed that increased energy density led to a softening behavior of the powder particles due to the extensive thermal energy, resulting also in an increase of the elastic modulus. Indicatively, P5 and P6 exhibited the highest elastic modulus, while both were manufactured with energy density above 150 J/mm^3^. The elastic modulus and the mechanical behavior were influenced by the applied thermal energy and its diffusion. This explains the fact that specimen P18 manufactured with energy density around 160 J/mm^3^ presented a low elastic modulus as the thermal energy was diffused to a large spot size (75 μm). The yield strength and the UTS followed similar behavior according to the alteration on the values of the applied AM conditions. The AM conditions that resulted in UTS values above 1050 MPa like P18, P15, and P24, demonstrated the best performance in terms of mechanical strength. All these specimens were manufactured with the maximum value of laser power (160 W) and an energy density between 145 J/mm^3^ and 160 J/mm^3^. The high laser power provided the necessary thermal energy to fully melt the feedstock material, avoiding in that way the appearance of fusion defects. Moreover, as is shown in specimens P3 and P9, high energy density could lead to reduced mechanical strength due to the extensively applied energy on the material structure. Furthermore, the spot size retains an impact on the mechanical strength. For P1 there was an increased elastic modulus and hardness due to the low value of the spot size, and similar behavior was observed for P10 and P19, which have lower VEDs but the same spot size (45 μm), resulting in elevated values of elastic modulus and hardness. However, the optimal mechanical behavior was observed, utilizing moderate values of spot size around 60 μm. Finally, it is worth mentioning that this macroscopic analysis was performed, taking into account that the size of the grains produced insignificant changes due to the relatively small range of applied thermal energy of the manufactured specimens [23,24].

### 3.4. Regression-Based Predictive Models

Second-order polynomial regression models were used to predict the behavior and the performance of the SLM AM procedure. More specifically, four models were formulated in order to compute the surface roughness, Young’s modulus, ultimate tensile strength, and yield strength in relation to the printing parameters of the SLM process. The efficiency of the developed models was measured by various estimators such as the mean absolute error (MAE), root mean squared error (RMSE), and the mean absolute percentage error (MAPE). These criteria were calculated through the following equations:

(9) $$\mathrm{MAE}=\frac{1}{N}\overset{N}{\underset{e=1}{\sum }}\left|y_{e}-\hat{y_{e}}\right|\;$$

(10) $$\mathrm{RMSE}=\sqrt{\frac{1}{N}\overset{N}{\underset{e=1}{\sum }}{\left(y_{e}-\hat{y_{e}}\right)}^{2}}\;$$

(11) $$\mathrm{MAPE}=\frac{100}{N}\overset{N}{\underset{e=1}{\sum }}\frac{\left|y_{e}-\hat{y_{e}}\right|}{\left|y_{e}\right|}\;$$

where N is the total number of the observations, y_e_ are the real observed values, and $\hat{y_{e}}$ are the estimated values computed from the regression models. It must be noted that MAPE is one of the most useful indications to evaluate the accuracy of forecasts, since it takes into account the relative performance of the model. Table 4 presents the results concerning the calculated coefficients for each of the developed regression models. More specifically, the computed coefficients b_0_,b_1_,..,b_33_ can be implemented in Equation (2) in order to calculate the investigated properties of the fabricated parts manufactured via the SLM process. In addition, the results of these equations were used to estimate the performance of the models. It must be noted that the total number of these coefficients is equal to c = 1 + 2n + n (n−1)/2 = 10, considering that the independent input variables are three. The performance for each of the regression-based models is exhibited by the means of MAE, RMSE, and MAPE in the bottom part of Table 4. In general, the computed results of the models are in good agreement with the experimental data with the maximum MAPE value equal to 8.83% in the case of the surface roughness model.

Furthermore, 10 additional experiments were conducted in order to further investigate the performance of the developed models. That validation set was applied in order to provide an unbiased evaluation of the developed regression models. It must be noted that these measurements were excluded during the development of the regression models and are utilized only for the validation stage of the models. The coefficients of each model presented in Table 4 were used to compute the corresponding properties of the fabricated parts using the SLM technology. The results are presented in Table 5 and albeit the mean absolute percentage errors between the predicted and the actual experimental values were higher compared to the calculated ones in Table 4, these deviations are relatively low with values smaller than 10% in all cases except from the surface finish model.

**Figure 8:**
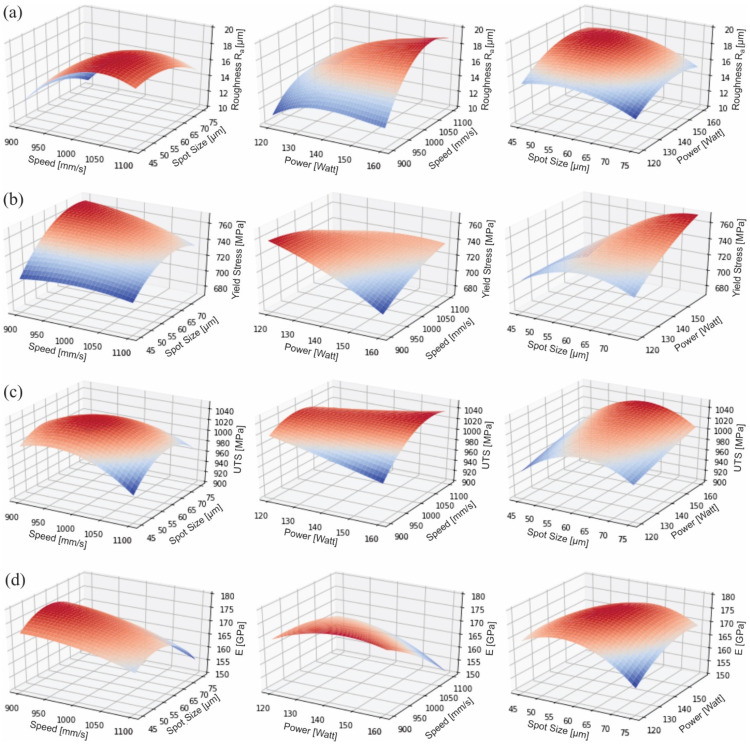
Response surface plots for the regression model of (**a**) Roughness, (**b**) Yield Strength, (**c**) Ultimate Tensile Strength and (**d**) Elastic Modulus dependent on the examined parameters.

Therefore, the developed regression models could be employed for estimating the mechanical behavior, as well as the surface roughness of metal, AM parts using the SLM technology and Inconel 718 as the feedstock material. In addition, the surface plots for the investigated models are illustrated in Figure 8. In order to create these plots, the regression coefficients of Table 4 were considered to calculate the corresponding values, i.e., surface roughness, Young’s modulus, ultimate tensile strength, and yield strength (Output data) depending on the printing conditions (input data). By inspecting the response surface, it is evident that the examined printing parameters interact and affect the output variables. More specifically, Figure 8a shows the 3D curves of the regression models for R_a_ related to the three examined process-related parameters. In the first two images, it is clear that the scan speed is the parameter that most influences the the surface quality, causing large deviations in the roughness values with changes in scan speed value. This phenomenon was expected due to the impact of scan speed on the surface tension of the molten pool. According to the literature [8], high scan speed leads to sufficient diffusion of thermal energy forming uniform molten pool and reducing the roughness. Laser power had a mild impact on roughness with the lowest roughness at medium values of laser power. As the laser power decreases, a lack of fusion defects appears, resulting in poor surface quality. In contrast, when the laser power rises, extensive thermal energy elicits intense surface tension in molten pools, increasing the roughness value. The spot size seems to operate as a diffuser of thermal energy in cases where the energy density is high, or there is low scan speed or high laser power, improving the surface quality.

The rest of the Figure 8b–d concerns regression models for the changes on mechanical properties, applying different process-related parameters. Yield strength and ultimate tensile strength have shown similar trends and 3D curves, according to Figure 8b,c. The performance and the quality of the printed parts using the SLM technology are directly dependent on the heat input that is mainly controlled by the employed printing conditions. In general, the liquid phase of the manufactured products is reliant on the melting temperature of the applied material, as well as on the energy that is transferred to the powder. The most dominant parameters affecting this energy are the scanning speed and laser power of the AM process. Laser power and scan speed are connected under the dependable variable of energy density, which shows if the parameters are suitable to provoke sufficient melting without overburning the employed material. Thus, when they are examined separately, no distinct pattern is observed. However, the coupling of these two parameters shows that in case these parameters do not follow the same trend, the strength of the specimens deteriorates and there are specific matches of scan speed and laser power values that improve the strength of the SLM AM parts. For example, when the scan speed is high, the laser power should be high in order to achieve notable mechanical strength, as is shown in the middle image of Figure 8c. Concerning the influence of the laser spot size, it should be large enough in order to provide sufficient energy density to melt the powder. However, it should be small enough in order to achieve the requirements of the surface finish. In our study, the spot size was observed to enhance the mechanical performance; more specifically, a spot size value at 60 μm improved all the examined mechanical properties (yield strength, UTS and elastic modulus). In addition, spot sizes lower or higher from the aforementioned value resulted in lower mechanical strength and a reduction of the elastic modulus. Furthermore, the elastic modulus rises when the laser power or energy density (low laser power and low scan speed) increases, due to the fact that the increased thermal strains arouse a more ductile mechanical response. It must be noted, that in situations where even larger spot sizes are applied, the mechanical behavior of the produced parts will deteriorate due to the lower generated density created from a large spot size. Finally, Figure 8, overall, provides accurate continuous trends of the examined quality factors, namely the surface quality and the basic mechanical response, exploiting the acquired experimental data and utilizing regression models. Therefore, it is possible to predict the final quality of Inconel 718 3D printed components when using the SLM method by just knowing the basic applied process-related parameters.

## 4. Conclusions

The current study investigates the influence of three major process-related parameters (the laser power, the scan speed, and the spot size) on the final quality of a metal AM part. For this purpose, Inconel 718 specimens were manufactured with various sets of parameters utilizing the SLM technology. The material characterization of the feedstock powder was performed with EDX, PSD, and morphological analyses to verify the quality of the raw material. The surface’s quality was evaluated by measuring the surface roughness and the mechanical performance of each specimen, which was achieved via nanoindentation and uniaxial tensile experiments. The results revealed that the scan speed has a severe impact on the surface quality, where low scan speeds lead to satisfactory surface finish. Furthermore, the laser power and the spot size presented a mild influence on the roughness, regulating the surface tension of molten pools through the absorption of thermal energy. Regarding the mechanical behavior, the energy density had an intense impact on the presence of defects in the part’s structure. Thus, by moderating the energy density i.e., by regulating the laser power, an enhanced mechanical performance on the 3D-printed parts was observed. It is also worth mentioning that the concentration or the diffusion of thermal strains during the manufacturing procedure could lead to the improvement or the reduction of the elastic modulus, respectively. Finally, regression-based models were developed that predict several mechanical properties as well as the surface roughness of parts manufactured via the SLM process using Inconel 718 superalloy as the feedstock material. It should be noted that with the aid of the developed models, it is feasible to select the values of the printing parameters that could result in the desired mechanical behavior. Considering all the above, a reduction of the AM production cost could be potentially achieved via the employment of suitable printing conditions in order to decrease the scrap parts and increase the efficiency of the manufacturing process itself.

## Figures and Tables

**Table 1:** Printing parameters of the SLM process for the training and validation set.

**Printing Parameters for the Training Set**					
		**Laser Power**			
		**120 W**	**140 W**	**160 W**	
Spot size	45 μm	P1	P2	P3	Scan speed = 900 mm/s
	60 μm	P4	P5	P6	
	75 μm	P7	P8	P9	
	45 μm	P10	P11	P12	Scan speed = 1000 mm/s
	60 μm	P13	P14	P15	
	75 μm	P16	P17	P18	
	45 μm	P19	P20	P21	Scan speed = 1100 mm/s
	60 μm	P22	P23	P24	
	75 μm	P25	P26	P27	
**Printing Parameters for the Validation Set**					
**Specimens ID**	**Laser Power (W)**		**Scan Speed (mm/s)**		**Spot Size (μm)**
P28	130		950		50
P29	130		950		55
P30	130		1050		65
P31	130		1050		70
P32	150		950		50
P33	150		950		55
P34	150		1050		65
P35	150		1050		70
P36	135		1025		52
P37	155		975		67

**Table 2:** Nominal weight percent of Inconel’s 718 chemical composition and its particle size distribution derived from the manufacturer.

**Weight Percent [%]**							
Ni	Cr	Fe	Nb + Ta	Mo	Al	Ti	Other
Balance	18	18	5	3	0.6	1	<0.5
**Particle Size Distribution**							
Nominal range		D90(μm)		D50(μm)		D10(μm)	
−45 + 15		46		30		18	

**Table 3:** Mechanical properties for wrought Inconel 718 samples [23,43] and the examined specimens.

Specimens	Energy Density (J/mm^3^)	Microhardness (MPa)	Elastic Modulus (GPa)	Yield Strength (MPa)	UTS (MPa)
Wrought	-	4750 [43]	200	916	1055
P1	133.33	3251 ± 473	170	750	1009
P2	155.56	2421 ± 236	163	650	990
P3	177.78	2256 ± 203	155	680	954
P4	133.33	3186 ± 143	136	770	1014
P5	155.56	3153 ± 306	234	750	1009
P6	177.78	3022 ± 242	194	700	1003
P7	133.33	2989 ± 254	184	735	990
P8	155.56	2432 ± 105	154	800	902
P9	177.78	2012 ± 181	183	730	839
P10	120.00	2995 ± 165	189	720	991
P11	140.00	2881 ± 274	179	680	967
P12	160.00	2756 ± 254	172	670	990
P13	120.00	2687 ± 242	154	740	972
P14	140.00	3135 ± 304	170	750	993
P15	160.00	3284 ± 296	185	740	1053
P16	120.00	2765 ± 249	163	780	1007
P17	140.00	2777 ± 250	163	780	1045
P18	160.00	3310 ± 298	146	760	1057
P19	109.09	2994 ± 269	170	715	1002
P20	127.27	2765 ± 205	152	700	955
P21	145.45	3075 ± 277	146	700	980
P22	109.09	2668 ± 240	152	760	1002
P23	127.27	2998 ± 270	169	745	1042
P24	145.45	3184 ± 287	160	750	1051
P25	109.09	2558 ± 230	168	670	933
P26	127.27	3017 ± 272	152	760	998
P27	145.45	2699 ± 243	167	770	953
P28	136.84	2610 ± 172	137	660	939
P29	136.84	2649 ± 204	173	695	953
P30	123.81	2727 ± 240	173	725	981
P31	123.81	2722 ± 177	164	710	979
P32	157.89	2694 ± 216	161	650	969
P33	157.89	2394 ± 215	166	680	861
P34	142.86	2758 ± 248	162	715	992
P35	142.86	2672 ± 214	171	690	961
P36	131.71	2597 ± 156	157	650	934
P37	158.97	2541 ± 102	159	700	914

**Table 4:** Polynomial regression coefficients and performance for each of the developed models considering the training data.

**Coefficient**	**Roughness**	**Young’s Modulus**	**Ultimate Tensile Strength**	**Yield Strength**
b_0_	−360.322	−521.863	−489.93	944.88
b_1_	0.4532	−10.015	4.278	−8.705
b_2_	0.5109	3.812	0.631	0.223
b_3_	0.8221	10.509	2.937	9.111
b_11_	−0.0033	−0.0032	−0.0095	−0.015
b_12_	0.00058	0.0101	−0.0023	0.009
b_13_	−0.00082	−0.00003	0.0118	0.059
b_22_	−0.00028	−0.0029	−0.00017	−0.00058
b_23_	−0.0001	0.0098	−0.00066	−0.0052
b_33_	−0.0056	−0.173	−0.033	−0.085
**Estimator**	**Roughness**	**Young Modulus**	**Ultimate Tensile Strength**	**Yield Strength**
MAE	1.29	28.02	11.91	15.8
RMSE	1.41	34.81	16.01	21.09
MAPE [%]	8.83	2.87	7.65	2.19

**Table 5:** Results for the performance of the regression models on the validation set.

**Exp. No.**	**Roughness Ra(μm)**		**Yield Strength (MPa)**		**UTS (MPa)**		**Young Modulus (GPa)**	
	**Meas.**	**Pred.**	**Meas.**	**Pred.**	**Meas.**	**Pred.**	**Meas.**	**Pred.**
P28	14.51	15.54	660	733.1	939	1020	137	175.4
P29	17.55	15.68	695	747.5	953	1028	173	177.2
P30	15.38	16.6	725	752.1	981	1024	173	169.9
P31	14.39	15.85	710	751.1	979	1012	164	166.4
P32	18.72	16.25	650	704.6	969	1007	161	176.4
P33	18.54	16.31	680	724.8	861	1015	166	179.4
P34	15.9	18.22	715	759.3	992	1033	162	169.9
P35	14.69	17.40	690	764.1	961	1020	171	167.5
P36	21.12	17.99	650	735	934	1025	157	173.3
P37	20.39	16.27	700	755.7	914	1010	159	177.1
**Estimator**	**Roughness**		**Yield Strength**		**UTS**		**Young’s Modulus**	
MAE	2.254		55.27		71.58		12.26	
RMSE	2.421		57.74		79.78		16.04	
MAPE [%]	12.9		8.12		7.70		7.99	

## Data Availability

Data sharing is not applicable.
